# Supplementary material for: Associations of Objectively-Measured Sedentary Time and Patterns with Cognitive Function in Non-Demented Japanese Older Adults: A Cross-Sectional Study
Source: Int J Environ Res Public Health. 2022 Feb 11;19(4):1999. doi: 10.3390/ijerph19041999 (PMC8871939; doi:10.3390/ijerph19041999)
Supplement: Supplementary file 1 [file ijerph-19-01999-s001.zip › ijerph-1548401-supplementary.pdf]

Supplementary materials

Supplementary Table S1. Associations between total sedentary time with performance in global cognitive function and specific cognitive domains (n = 1,681)

	β (95% CI)				
**Cognitive functions**	Quartile 1 (low)	Quartile 2	Quartile 3	Quartile 4 (high)	P value for trend
**Total MoCA score**					
Age- and sex-adjusted	reference	0.06 (-0.05 to 0.18)	0.06 (-0.07 to 0.18)	0.12 (-0.01 to 0.24)	0.11
Multivariable adjusted*	reference	0.06 (-0.05 to 0.18)	0.08 (-0.04 to 0.19)	0.09 (-0.04 to 0.22)	0.16
Additionally adjusted for MVPA†	reference	0.09 (-0.03 to 0.21)	0.12 (-0.01 to 0.25)	0.15 (0.01 to 0.30)	0.04
**Orientation**					
Age- and sex-adjusted	reference	-0.02 (-0.10 to 0.13)	-0.02 (-0.14 to 0.09)	-0.04 (-0.17 to 0.08)	0.39
Multivariable adjusted*	reference	0.00 (-0.11 to 0.12)	-0.03 (-0.15 to 0.09)	-0.07 (-0.20 to 0.05)	0.23
Additionally adjusted for MVPA†	reference	0.02 (-0.09 to 0.14)	0.00 (-0.13 to 0.13)	-0.03 (-0.17 to 0.11)	0.60
**Visuospatial abilities**					
Age- and sex-adjusted	reference	-0.04 (-0.17 to 0.09)	-0.03 (-0.16 to 0.10)	-0.04 (-0.18 to 0.10)	0.64
Multivariable adjusted*	reference	-0.04 (-0.17 to 0.09)	-0.01 (-0.14 to 0.12)	-0.04 (-0.19 to 0.10)	0.63
Additionally adjusted for MVPA†	reference	-0.03 (-0.16 to 0.11)	0.01 (-0.14 to 0.15)	-0.03 (-0.19 to 0.14)	0.90
**Short-term memory**					
Age- and sex-adjusted	reference	0.04 (-0.08 to 0.17)	0.04 (-0.08 to 0.17)	-0.04 (-0.17 to 0.09)	0.62
Multivariable adjusted*	reference	0.05 (-0.08 to 0.17)	0.04 (-0.08 to 0.17)	-0.03 (-0.17 to 0.10)	0.64

Additionally adjusted for MVPA†	reference	0.05 (-0.07 to 0.18)	0.05 (-0.08 to 0.18)	-0.02 (-0.16 to 0.12)	0.83
**Executive function**					
Age- and sex-adjusted	reference	0.10 (-0.03 to 0.23)	0.09 (-0.04 to 0.22)	0.12 (-0.02 to 0.26)	0.14
Multivariable adjusted*	reference	0.09 (-0.04 to 0.22)	0.08 (-0.05 to 0.22)	0.07 (-0.07 to 0.22)	0.36
Additionally adjusted for MVPA†	reference	0.09 (-0.04 to 0.23)	0.08 (-0.06 to 0.23)	0.07 (-0.09 to 0.23)	0.45
**Attention, concentration and working memory**					
Age- and sex-adjusted	reference	0.10 (-0.03 to 0.22)	0.08 (-0.05 to 0.20)	0.17 (0.04 to 0.31)	0.02
Multivariable adjusted*	reference	0.10 (-0.02 to 0.22)	0.10 (-0.02 to 0.23)	0.17 (0.03 to 0.31)	0.02
Additionally adjusted for MVPA†	reference	0.12 (-0.01 to 0.25)	0.14 (0.00 to 0.27)	0.22 (0.06 to 0.37)	0.01
**Language**					
Age- and sex-adjusted	reference	0.04 (-0.09 to 0.16)	0.03 (-0.10 to 0.16)	0.12 (-0.02 to 0.26)	0.12
Multivariable adjusted*	reference	0.04 (-0.09 to 0.16)	0.04 (-0.09 to 0.17)	0.11 (-0.03 to 0.25)	0.15
Additionally adjusted for MVPA†	reference	0.06 (-0.07 to 0.19)	0.07 (-0.07 to 0.21)	0.15 (-0.01 to 0.31)	0.08

Note: CI, confidence interval; MVPA, moderate-vigorous physical activity. The quartile cut-points for total sedentary time were -77, 0, and 74 (for the categorization of quartiles, values of total sedentary time were adjusted for accelerometer wear time by using the residuals method).

* Adjusted for age (continuous) and sex (men or women), years of educations (continuous), living alone (yes or no), body mass index (continuous), multimorbidity (yes or no), employed (yes or no), current smoker (yes or no), current drinker (yes or no).

† MVPA was adjusted for accelerometer wear time by using the residuals method.

Supplementary Table S2. Associations of prolonged sedentary time and mean sedentary bout length with impairment in the domain of orientation ability (n = 1,681)

	Number of participants	Odds ratio (95%CI)		
		Age- and sex-adjusted	Multivariable adjusted*	Multivariable model additionally adjusted for MVPA†
**Prolonged sedentary time in bouts of ≥ 30 minutes**				
Quartile 1 (low)		reference	reference	reference
Quartile 2		1.29 (0.78 to 2.13)	1.33 (0.80 to 2.22)	1.32 (0.79 to 2.20)
Quartile 3		1.44 (0.88 to 2.36)	1.50 (0.91 to 2.48)	1.32 (0.87 to 2.49)
Quartile 4 (high)		1.93 (1.17 to 3.16)	2.07 (1.24 to 3.45)	2.02 (1.17 to 3.48)
P value for trend		0.008	0.004	0.01
**Average sedentary bout length**				
Quartile 1 (low)		reference	reference	reference
Quartile 2		0.82 (0.51 to 1.34)	0.84 (0.51 to 1.38)	0.83 (0.50 to 1.36)
Quartile 3		1.00 (0.62 to 1.60)	1.01 (0.63 to 1.63)	0.98 (0.60 to 1.59)
Quartile 4 (high)		1.58 (1.01 to 2.49)	1.61 (1.01 to 2.57)	1.54 (0.95 to 2.49)
P value for trend		0.02	0.02	0.04

Note: Impairment in the domain of orientation ability was defined as the orientation score < 6 points (i.e., less than the total orientation score). CI, confidence interval; MVPA, moderate-vigorous physical activity. The quartile cut-points for prolonged sedentary time were -78,

-18, and 58 (for the categorization of quartiles, values of prolonged sedentary time were adjusted for accelerometer wear time by using the residuals method). The quartile cut-points for mean sedentary bout length were 6.1, 7.6, and 9.5 min.

* Adjusted for age (continuous) and sex (men or women), years of educations (continuous), living alone (yes or no), body mass index (continuous), multimorbidity (yes or no), employed (yes or no), current smoker (yes or no), current drinker (yes or no).

† MVPA was adjusted for accelerometer wear time by using the residuals method.
